# N-Acetyl Cysteine Ameliorates High-Fat Diet-Induced Nonalcoholic Fatty Liver Disease and Intracellular Triglyceride Accumulation by Preserving Mitochondrial Function

**DOI:** 10.3389/fphar.2021.636204

**Published:** 2021-09-13

**Authors:** Weijian Hang, Hongyang Shu, Zheng Wen, Jinyan Liu, Zhiyuan Jin, Zeqi Shi, Chen Chen, Dao Wen Wang

**Affiliations:** ^1^ Division of Cardiology, Department of Internal Medicine, Tongji Hospital, Tongji Medical College, Huazhong University of Science and Technology, Wuhan, China; ^2^ Hubei Key Laboratory of Genetics and Molecular Mechanisms of Cardiological Disorders, Wuhan, China

**Keywords:** NAC, NAFLD, mitochondria, PGC1a, SIRT1

## Abstract

**Rationale:** Nonalcoholic fatty liver disease (NAFLD) is a kind of metabolic disease characterized by liver steatosis. Excessive reactive oxygen species (ROS) originating from dysfunctional mitochondria is the major pathophysiological contributor in the development of NAFLD and is thought to be a promising therapeutic target. A few reports demonstrate the antioxidative treatments for NAFLD.

**Methods:** Male C57 mice were fed on a normal chow diet (ND) or high-fat diet (HFD) for 8 weeks. PBS or N-acetyl cysteine (NAC) was gavaged to mice. LO2 human liver cell line treated with palmitic acid (PA) was applied as a cellular model. Western blot, immunofluorescence, biochemistry assay, and pathological staining were used to investigate the mechanism of suppressing lipid accumulation of NAC.

**Results:** NAC treatment was able to prevent HFD-induced NAFLD, as evidenced by less hepatic triglyceride accumulation and lipid droplet formation compared with that of mice in the HFD group. NAC could preserve mitochondrial function by inhibiting excessive mitophagy and promoting mitochondria biogenesis to prevent ROS production. NAC also activated Sirt1 and preserved its protein level and subsequently promoted mitochondria biogenesis *via* deacetylating PGC1a.

**Conclusion:** We demonstrated that NAC may be an effective drug to treat NAFLD, which was related to its antioxidative and mitochondrial protective effect.

## Introduction

Nonalcoholic fatty liver disease (NAFLD) is characterized by hepatic steatosis and hepatic dysfunction (Wang and Malhi, 2018). Changes in lifestyle, especially excessive lipid intake in daily diet, lead to a higher prevalence rate of NAFLD in China as well as worldwide (Zhou et al., 2019). NAFLD will progress into nonalcoholic steatohepatitis (NASH) and eventually into hepatocellular carcinoma (HCC), which needs to pay much attention to early interference and improve prognosis (Mohamad et al., 2016; Liu and McCaughan, 2018).

The provoking character of NAFLD is the abnormality of lipid metabolism, leading to the accumulation of excessive lipid, especially triglyceride (TG). As the major cell organelle to catabolize TG, mitochondrion is reported to display an important pathophysiological role in the development of NAFLD (Paradies et al., 2014; Bessone et al., 2019). Mitochondrial dysfunction is characterized by reduced mitochondrial quantity, compromised mitochondrial function, and abnormal mitochondrial morphology and is related to various human diseases including metabolism disorder (Mansouri et al., 2018). Mitochondrial dysfunction can intrinsically activate mitochondria biogenesis to refresh the intracellular mitochondria pool, which is tightly related to PGC1a, the central mitochondria biogenesis transcriptional coactivator (Yeo et al., 2019). PGC1a can be reduced under metabolism disorder and result in disturbed mitochondria biogenesis (Santos et al., 2011; Chen et al., 2017), and it was reported that preserving PGC1a activity could effectively protect against HFD-induced diabetic cardiomyopathy (Waldman et al., 2018). Hence, PGC1a can be a promising target in treating metabolic dysfunctions including NAFLD.

As a central metabolic cellular organelle, mitochondrial function is under rigorous regulation by several signaling pathways, including Sirtuin (silent mating type information regulation 2 homolog-1) pathway. Sirtuins are an important family of deacetylases and are very conservative among mammals (de Gregorio et al., 2020). Sirtuins family consists of seven members, from Sirt1 to Sirt7, and Sirtuins’ activity is tightly regulated by intracellular NAD^+^/NDAH ratio (Tang, 2016). Sirt1 is reported to be an important energy sensor and is involved in increasing healthspan and lifespan (Ding et al., 2017). As an important deacetylase, Sirt1 is involved in the posttranslation regulation of targeted proteins including PGC1a. The disturbance of Sirt1/PGC1a signaling pathway is involved in several pathological conditions such as ischemia-reperfusion injury (Tang et al., 2019) and aging (Kalfalah et al., 2014). Moreover, Sirt1/PGC1a is tightly related to metabolism dysfunctions such as diabetic cardiomyopathy (Waldman et al., 2018) and diabetic kidney injury (Hong et al., 2018). It is hence very promising to find an effective strategy to retain normal Sirt1/PGC1a signaling in metabolism disorders such as NAFLD.

Mitochondrial dysfunction usually results in excessive production of reactive oxygen species (ROS), which can contribute to several diseases including NAFLD (Simões et al., 2018). Hence, one of the promising therapies is to reduce the production of ROS and to maintain the normal function of mitochondria. As a widely applied antioxidant agent, N-acetyl cysteine (NAC) is usually considered as ROS scavenger in basic experimental investigations (Rushworth and Megson, 2014) and is demonstrated to be effective in treating murine diseases such as ischemia/reperfusion injury (Wang et al., 2014a) and lung cancer (Sayin et al., 2014). However, in clinical work, it is usually used as a mucolytic agent (Li et al., 2018a) and prototypical antidote administered after an acetaminophen overdose, and little attention has been paid to its antioxidant pharmacological property (Rushworth and Megson, 2014). Recently, several limited clinical trials indicated that NAC may be effective in attenuating hepatic dysfunction and ameliorating NAFLD (Khoshbaten et al., 2010). However, the exact mechanism(s) underlying this clinical effect is far from being fully uncovered. Hence, we hereby report that NAC showed a protective effect against the high-fat-diet-induced mouse NAFLD model *via* protecting mitochondria function and improving mitochondria quantity by preserving the Sirt1/PGC1a signaling pathway.

## Material and Method

### Animal

All animal experiments were performed according to the Experimental Animal Care and Use Committee of Tongji Medical College, Huazhong University of Science and Technology. Male C57Bl/6 mice aged around 8–10 weeks and weighted 25–27 g were kept at SPF experimental animal environment at Experiment Animal Center. Mice were kept, five mice per cage, under a standard housing environment with a 12 h light/12 h dark cycle and have access to water and food ad libitum.

### Experiment Design

After 1 week of preadaptation fed, C57 mice were divided randomly into different groups: normal diet (ND), high-fat diet (HFD), NAC gavage (NAC), and high-fat diet with NAC (HFD + NAC). 150 mg/kg NAC were gavaged daily 1 week prior to HFD and lasted for the following 8 weeks of HFD treatment. After 1 week of NAC treatment, mice were then fed with a HFD (Research Diet, D12492, with 60% Kcal of fat, 20% Kcal of protein, and 20% Kcal of carbohydrate) or a normal chow diet (ND) for 8 weeks. Every 2 weeks, blood samples were collected from the tail vein and used for blood chemistry test (Jiancheng Biotechnology Institution, Nanjing) according to the manufacturer’s protocols. At the endpoint of the 8 weeks, mice were harvested and liver tissues were obtained for further test.

For the test of the treatment effect of NAC, after 1 week of preadaptation, C57 mice were firstly fed with ND or HFD for 8 weeks. After 8 weeks of HFD, 150 mg/kg NAC were gavaged daily for the next 4 weeks. At the end of the 12 weeks, mice were harvested and liver tissues were obtained for further test.

### Glucose Tolerance Test

C57Bl/6 mice were fasted overnight (>8 h). Fasted blood glucose level was first measured, and then 50% D-glucose (w/v) were injected intraperitoneal at 4 ul/g (v/body weight). Blood glucose level was measured by OneTouch^®^ UltraVue (Johnson & Johnson) at 0 min (fasted blood glucose), 30, 60, 90, and 120 min through the tail vein. Each group had 5−8 mice.

### Adipocyte Cross-Section Area Measurement

White adipose tissue was fixed in 4% paraformaldehyde overnight and embedded in paraffin, and 5 μm sections were made for hematoxylin-eosin staining. After staining, each section was taken photo under a magnification of ×400. About 20 adipocytes in each section were randomly chosen, the cross-section area was calculated by ImageJ, and over 400 adipocytes were cumulatively analyzed in each group. Then, according to their cross-section area, the proportion of different cross-section areas was calculated.

### Cell Culture and Palmitic Acid (PA) Stimulation

LO2/7702 human liver cell line was purchased from Shanghai Cellbank of Chinese Academy of Sciences and were maintained with DMEM high glucose (Hyclone) with 10% FBS (Gibco) and 1% P/S, under the condition of 5% CO2. Cells were passed every 2 days, each colony of the cell was used for 4 weeks, and then a new colony would be revived. 5 × 10^6^ cells were seeded to one well of a 6-well plate or a confocal dish for different treatments. 2 × 10^7^ cells were seeded to one 10 cm dish for immunoprecipitation. PA was dissolved in BSA as previously reported (Li et al., 2019; Zhou et al., 2020), and a stock solution of 20 mM PA was made.

### Protein Extraction and Immunoblotting

Protein was extracted by RIPA cell lysis buffer. Briefly, approximately 10^6^ cells were lysed by 100 ul RIPA or liver tissue with a ratio of 1:20 tissue (mg): RIPA (ul), with tissue homogenizing. All RIPA were supplied with 1 mM PMSF, 1X Protease Inhibitor Cocktail (MCE), and 1X Phosphatase Inhibitor Cocktail (MCE), and all the processes were done on the ice. After lysis, all samples were subjected to centrifugation at 16,000 *g*, 4°C for 15 min. Supernatants were obtained and measured for protein concentration with BCA protein assay kit (Beyotime), followed by boiling samples with 5X protein loading buffer (Servicebio) at 100°C for 10 min.

For immunoblotting, 20–40 ug of total protein sample was subjected to 10–15% SDS-PAGE assay, and then the gel proteins were transferred to 0.45 um PVDF membrane (GE Health) with 1X transfer buffer supplied with 15% methanol at 100 V for 1–3 h, according to the protein molecular mass. After blocking the membrane with 5% skim milk TBST for 1 h and washing for three times, the membrane was incubated with an interested primary antibody at 4°C overnight. Then the membrane was incubated with HRP-conjugated secondary antibody and exposed by ECL kit. The following primary antibodies were used in this research: anti-MFF (CST, 84580), anti-Drp1 (CST, 8570), anti-VDAC (Abclonal, A19707), anti-Actin (Abclonal, AC026), anti-GAPDH (Abclonal, AC002), anti-PINK1 (Abclonal, A11435), anti-Parkin (Abclonal, A0968), anti-PGC1a (Abclonal, A11971), anti-Histone H3 (Abclonal, A2348), anti-acetylated lysine (Abclonal, A2391), anti-SOD1 (Abclonal, A0274), anti-SOD2 (Abclonal, A1340), anti-SREBP1c (Abclonal, A15586), anti-SREBP2 (Abclonal, A13049), anti-LPL (Abclonal, A16252), anti-NOX1 (Abclonal, A12309), anti-NOX2 (Abclonal, A12430), anti-NOX4 (Abclonal, A11274), and Sirtuin antibody sample kit (anti-Sirt1-7, CST, 9787).

### Immunohistochemistry and Immunofluorescence

Liver tissue samples of C57Bl/6 mice were fixed in 4% paraformaldehyde overnight and embedded in paraffin, and 5 μm sections were made for downstream immunohistochemistry staining. The sections were dewaxed and retrieved with TE buffer (pH 9.0). Freshly prepared 3% H_2_O_2_ was used to block intrinsic peroxidase. 5% BSA in 0.1% Triton X-100 PBS was used to block unspecific signal, and the sections were incubated with VDAC primary antibody (Abclonal, A19707) or NOX4 primary antibody (Abclonal, A11274) with 1:200 dilution at 4°C overnight. HRP-conjugated secondary antibody was incubated at room temperature for 50 min, and then DAB was used to reveal positive signals. The nucleus was counterstained by hematoxylin. Then images were taken with microscopy (CIC, SXP-C204) under a magnification of × 200.

As for cell immunofluorescence, the protocol was similar to the mentioned above, but with some modifications: cells were fixed with 4% paraformaldehyde at room temperature for 5 min and then permeated with 0.1% Triton X-100 at room temperature for 10–15 min. After blocking with 5% BSA in 0.1% Triton X-100 PBS, cells were incubated by PGC1a primary antibody (Abclonal, A11971) with 1:200 dilution at 4°C overnight. Then Cy3-conjugated secondary antibody was applied, while the nucleus was stained by DAPI. Then images were taken by Nikon confocal microscopy under a magnification of × 1,000.

### Mitochondrion DNA (mtDNA) Copy Number Quantification

mtDNA copy number quantification was carried based on the RealTime PCR (Jiang et al., 2017). Briefly, liver total DNA was purified with Total DNA Extraction Kit (Sangon, Shanghai) and quantified by NanoDrop. Then mtDNA specific gene mtND4 was amplified with the following primers: F: CTC​CTC​AGT​TAG​CCA​CAT​AGC​A; R: TGT​GGA​TCC​GTT​CGT​AGT​TGG​A (for *mus musculus*). 28SrRNA were used as internal control with the following primers: F: AGG​ACC​CGA​AAG​ATG​GTG​AAC​TA; R: CGG​AGG​GAA​CCA​GCT​ACT​AGA​T. 25 ng total DNA was amplified with qPCR SYBR-Green Master Mix (Yeasan, Shanghai) by BioRad FX96, and the relative copy number of mtDNA was measured by 2^−ΔΔCt^.

### Mitochondria Morphology Analysis

Cell mitochondria were stained by MitoTracker-Red (Invitrogen) at 37°C for 30 min and then washed by warmed-up PBS for two times. Photos of cell mitochondria were taken by Nikon confocal microscopy at 594 nm exciting laser with 1,024 × 1,024-bit rate. The length of mitochondria was measured by ImageJ in each cell, and about 20–30 cells per group were measured.

### Mitochondria Membrane Potential (MMP) Measurement

MMP was measured by the fluorescence intensity of TMRE (Invitrogen). Briefly, cells were incubated with 100 nM TMRE at 37°C for 30 min and then washed by warmed-up PBS for two times. Then cells were observed by Nikon fluorescence microscopy at Cy3 filter and photos were taken. The exciting light intensity was kept constant between each group. A median of five fields per sample was counted under a magnification of × 100, and each group had three samples. The fluorescence intensity of each picture was measured by ImageJ.

### ROS Measurement

Cell ROS level was measured by ROS measurement kit (Beyotime) according to the manufacturer’s protocol. Briefly, after proper treatment(s), the medium was discharged and cells were washed by PBS 3 times. Then ROS probes were loaded into cells at 37°C for 30 min in an incubator cage. After probes were loaded, cells were washed by warmed-up PBS 2 times to remove excessive probes. Then cells were observed by Nikon fluorescence microscopy at FITC filter and photos were taken. The exciting light intensity was kept constant between each group. A median of five fields per sample was counted under a magnification of × 100, and each group had three samples. The fluorescence intensity of each picture was measured by ImageJ.

### Cell TG Quantification

5 × 10^4^ cells were cultured at a 24-well plate. After proper treatments, cells were washed by chilled PBS 2 times and detached from the culture plate by 0.25% trypsin digestion. Cells were lysed by 0.5% NP-40 PBS and a frozen-thaw cycle was performed two times. Then the cell lysis was vortex and TG and protein levels were measured by TG quantification kit (Jiancheng Biotechnology Institution, Nanjing) and BCA Protein quantification kit (Beyotime). Cell TG level was adjusted to protein amount and is shown as mM (TG)/mg (Protein).

### Nuclear-Cytoplasm Protein Separation

Cell nuclear and cytoplasm protein were separated by Cell Nuclear-Cyto Separation Kit (Beyotime) according to the manufacturer’s guidelines. After separation, protein concentration was measured by the BCA protein quantification kit (Beyotime). 15 ug protein was loaded to SDS-PAGE analysis just like mentioned above. GAPDH and HistoneH3 were used as cytoplasm and nuclear loading control.

### Measurement of Cellular NAD^+^ and NADH and Measurement of Serum MDA

After collecting serum of mice, serum MDA was measured by malondialdehyde (MDA) assay kit (Nanjing Jiancheng Bioengineering Institute, A003-1-2) according to manufacturer’s protocol.

Cellular NAD^+^ and NADH level was quantified by NAD^+^/NADH Assay Kit with WST-8 (Beyotime, S0175) according to manufacturer’s protocol. The amount of NADH and the total amount of NADH plus NAD^+^ were measured separately, and the difference between them was calculated as the amount of NAD^+^. The ratio of NAD^+^/NADH was then calculated.

### siRNA Transfection

*Homo sapiens* PGC1a siRNA was purchased from Santa Cruz (sc-38884). 4 ul siRNA was transfected by 4 ul Lipo 2000 (Invitrogen) to 1 well of 6-well cell culture plate. 1 × 10^6^ cells were seeded to 1 well of a 6-well plate for siRNA transfection. After 48 h of transfection, cells were harvested to detect knockdown efficacy or for further experiments.

### Statistical Analysis

In our study, statistical analysis was carried out with GraphPad Prism 6. Data were expressed as means ± SD. The statistical significance of differences was determined by a two-way analysis of variance (ANOVA).

## Result

### NAC Ameliorated HFD-Induced Hepatic Steatosis and Metabolic Disturbance

**FIGURE 1:**
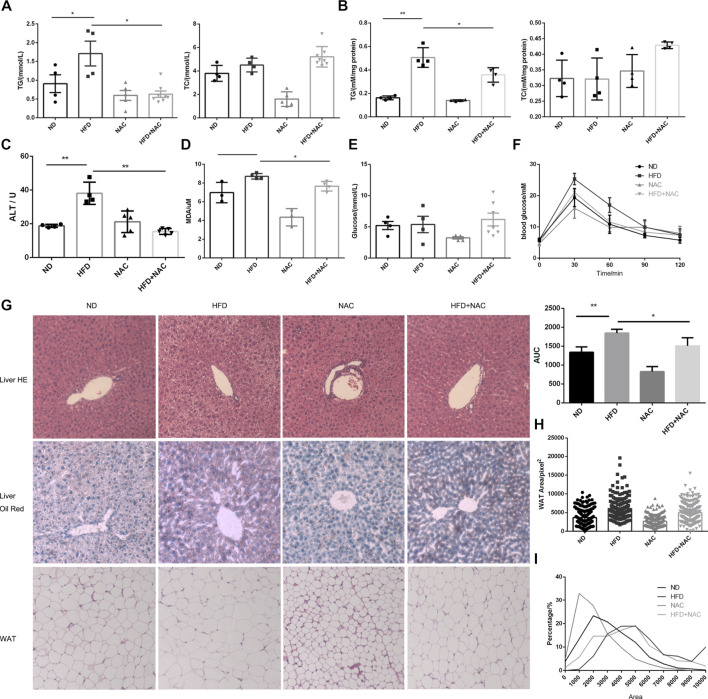
NAC treatment could alleviate HFD-induced NAFLD *in vivo*. **(A)** Concentration of TG and TC in serum of mice; **(B)** concentration of TG and TC in mice hepatic tissues, which was adjusted to protein concentration; **(C)** serum ALT level of mice; **(D)** concentration of MDA in serum of mice; **(E)** fasted blood glucose level; **(F)** blood glucose concentration of mice at 0, 30, 60, 90, and 120 min after oral glucose gavage, and the under curve area was calculated; **(G)** HE staining and oil red staining of section of live tissue and HE staining of mice white adipose tissue (WAT); **(H)** cross-section area of white adipocyte in different groups; **(I)** percentage of adipocyte with different cross-section areas (^*^ *p* < 0.05, ^**^ *p* < 0.01, and ^****^ *p* < 0.0001; all the data significance was analyzed by ANOVA).

We first tested whether NAC could ameliorate hepatic steatosis and hyperlipidemia *in vivo*. Male C57 mice were fed with a ND or HFD containing 60% fat for 8 weeks, while NAC was given 1 week prior to HFD and lasted for the following 8 weeks. Serum and hepatic TG levels were significantly elevated after HFD. However, little change in total cholesterol (TC) was detected (Figures 1A,B). Surprisingly, NAC showed a dramatic effect in decreasing TG level both in serum and in liver (Figures 1A,B). Significant elevation of alanine aminotransferase (ALT) was observed in the HFD group, while NAC could reverse the elevation of ALT, indicating the normalization of hepatic function (Figure 1C).

We also determined serum level of malondialdehyde (MDA), a marker of oxidative stress, and found that HFD indeed augmented the serum level of MDA significantly, while NAC treatment slightly but significantly mitigated MDA level (Figure 1D), which indicated the amelioration of oxidative stress.

It was reported that HFD can ultimately induce elevation of blood glucose level and cause diabetes in the rodent model. However, after testing fasting blood glucose, no difference between HFD and ND mice was found (Figure 1E). We hypothesized that it may be related to the different time periods of HFD treatment. Although no difference between fasting blood glucose levels was detected, results from the oral glucose tolerance test (OGTT) revealed impaired glucose tolerance. Interestingly, after NAC treatment, mice showed improved glucose tolerance as the area under curve (AUC) of OGTT was significantly reduced (Figure 1F). These results indicated that NAC could attenuate HFD-induced metabolic disturbance.

Hepatic steatosis was observed in HE staining of HFD mice liver section and NAC totally restored the normal morphology of hepatic tissue (Figure 1G). Enormous lipid droplets can be observed in the HFD mice liver section, while the lipid droplet accumulation was attenuated after NAC treatment (Figure 1G).

We have already shown that HFD can cause hypertriglyceridemia (Figure 1A). White adipose tissue (WAT) is the main organ to store excess lipid in the body; hence, we analyzed the cross-section area of adipocyte in WAT of different groups (Figure 1G) and found that, after HFD treatment, white adipocyte cells significantly turned larger than those of ND mice while NAC significantly reduced cross-section area of WAT adipocyte (Figure 1H). The proportion of larger adipocytes was higher in the HFD group than that in the ND group, and NAC reduced the proportion of larger adipocytes (Figure 1I). Taken together, these results revealed that NAC can effectively ameliorate HFD-induced hepatic steatosis *in vivo*.

### NAC Prevented TG Accumulation *via* Persevering Mitochondrial Function

**FIGURE 2:**
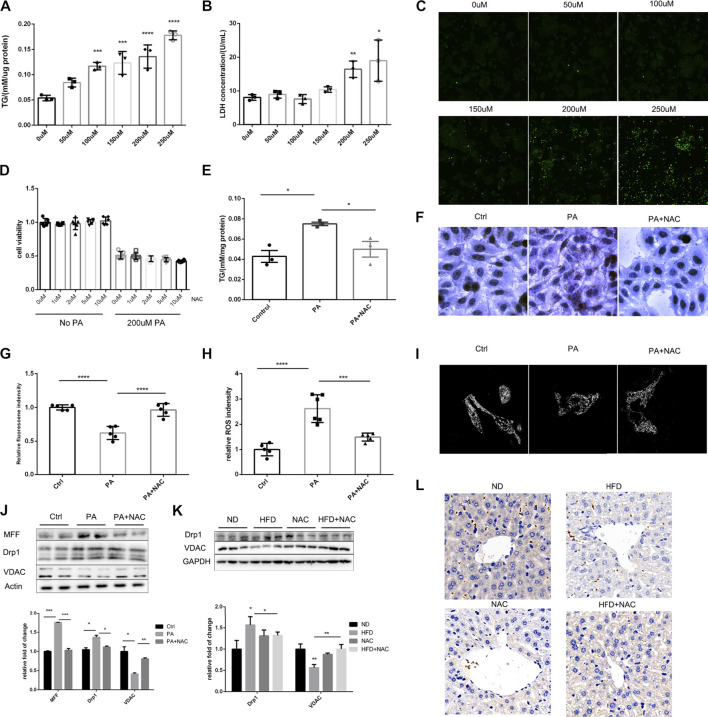
NAC treatment could reduce intracellular TG accumulation *via* preserving mitochondrial function. **(A)** Intracellular TG content was measured with TG measurement kit, and the concentration was adjusted to protein level; **(B)** LDH releasement in cell culture medium under different PA concentration stimulation; **(C)** DHE staining showed intracellular ROS level under different PA concentration; **(D)** cell viability of different concentration of NAC treatment under control and 200 μM PA stimulation; **(E)** intracellular TG concentration after PA stimulation and NAC treatment; **(F)** oil red staining of LO2 cell stimulated with PA or together with NAC treatment; **(G)** mitochondrial membrane potential of LO2 cells measured by TMRE fluorescence intensity in each group; **(H)** intracellular ROS level measured by DHE fluorescence intensity in each group; **(I)** LO2 cells were stained by MitoTracker and its mitochondrial morphology was observed by confocal microscopy. More fragmented mitochondria were observed in the PA group; **(J,K)** representative Western blot image of mitochondria fission-related proteins after different treatment in LO2 cells **(J)** or in mice hepatic tissue **(K)**; the experiment was conducted triple; **(L)** immunohistochemistry analysis of mice hepatic VDAC protein level (^*^ *p* < 0.05, ^**^ *p* < 0.01, ^***^ *p* < 0.001, and ^****^ *p* < 0.0001; all the data significance was analyzed by ANOVA).

As was reported by several groups, PA treatment can mimic hyperlipidemia *in vitro* (Li et al., 2019; Zhou et al., 2020). Hence, different concentration of PA was added to the medium of LO2 cell, and we found that the intracellular TG level showed a positive dose-dependent relationship with PA concentration (Figure 2A). We also determined LDH level in cell culture medium, a classical cell injury marker. LDH was significantly elevated when the concentration of PA was over 200 μM (Figure 2B). DHE probe was used to detect intracellular ROS, and intracellular ROS was obviously upregulated with the elevation of PA concentration (Figure 2C). Hence, we next applied 200 μM PA treatment to mimic HFD-induced cell injury *in vitro*.

Although NAC treatment cannot improve cell viability after PA treatment, it did not further deteriorate the cell viability (Figure 2D), suggesting its safety in future translational researches. NAC was able to ameliorate intracellular TG accumulation as proven by intracellular TG quantification (Figure 2E) and cell oil red staining (Figure 2F). These results were consistent with the effects of NAC *in vivo*, which indicates this is an appropriate cell model.

It was well-recognized that mitochondrion is the main organelle to catabolize intracellular triglyceride; hence, we evaluated mitochondrial function by TMRE (tetramethylrhodamine ethyl ester), a dye that can monitor MMP, and MitoSOX, which can indicate the level of mitochondrial ROS. As shown in Figures 2G,H, PA treatment reduced MMP and elevated mitochondrial ROS, while NAC treatment could totally restore mitochondrial function. Considering that NAC is a well-established antioxidant reagent, we measured intrinsic hepatic oxidases including SOD1 and SOD2. Although HFD did not significantly reduce the hepatic expression level of SOD1 and SOD2, NAC supplement did increase hepatic SOD1 (Supplementary Figure S1A), indicating enhanced intrinsic antioxidant capacity. Another important concern is about the resource of ROS, which was reported to be related to NOXs. We measured NOX1, NOX2, and NOX4 in mice livers and found no significant differences (Supplementary Figures S1C,D).

We also observed mitochondrial morphology by confocal microscopy and found that, after PA treatment, mitochondrion turned to be more fragmental and shorter compared to normal cells, whose mitochondrion showed tubular morphology. Interestingly, NAC could also restore the normal morphology of mitochondrion, which could partially explain the reason for its ability to restore mitochondrial function, as it is widely acknowledged that mitochondrion morphology shows consistency with its function (Youle and van der Bliek, 2012).

After observing significant fragmental mitochondria, we determined the protein level of MFF and Drp1, which were responsible for mitochondrion fission. It was revealed that PA treatment significantly upregulated the expression of MFF and Drp1, and NAC reversed the upregulation of these two proteins (Figure 2J). This may explain the improvement of mitochondrial morphology after NAC treatment. It was interesting to observe that the expression of VDAC, a housekeeping protein of mitochondrion, was well preserved by NAC treatment, while PA stimulation significantly downregulated the expression level of VDAC (Figure 2J). Similar results were also found in mice livers by Western blot (Figure 2K) and immunochemistry histological staining of VDAC (Figure 2L).

Taken together, these data indicated that NAC can attenuate triglyceride accumulation both *in vivo* and *in vitro*, and the lipid-lowering effect of NAC was related to the preservation of mitochondrial function, which needs further investigation.

### NAC Protected Mitochondria Function *via* Maintaining Mitochondria Homeostasis

**FIGURE 3:**
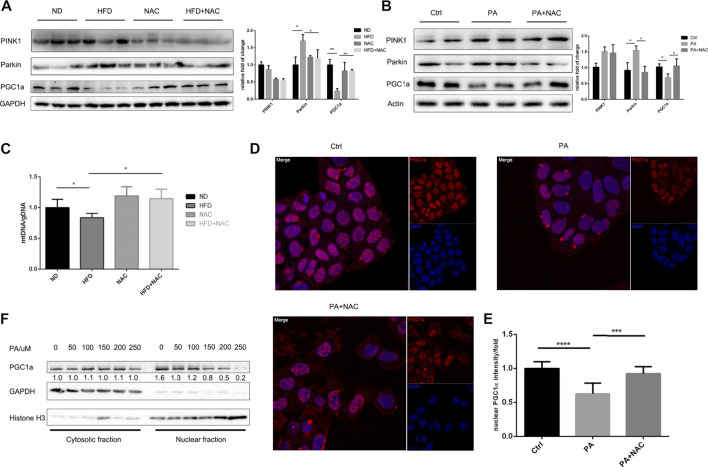
NAC treatment inhibits PA-induced mitophagy and promoted mitochondria biogenesis *via* PGC1a. **(A,B)** Representative Western blot image of mice hepatic tissue **(A)** and LO2 cell **(B)** revealed protein level of mitophagy initiating protein PINK1 and Parkin and mitochondria biogenesis key protein PGC1a. The experiment was conducted triple; **(C)** mitochondrial gDNA copy number in a different group of mice liver; **(D)** immunofluorescence of PGC1a revealed different subcellular localization, scale bar was 10 μm, and the nuclear PGC1a fluorescence intensity was analyzed in **(E)**; **(F)** separation of nuclear and cytosolic protein under different concentration of PA stimulation and protein level of PGC1a measured by Western blot (^*^ *p* < 0.05, ^***^ *p* < 0.001, and ^****^ *p* < 0.0001; all the data significance was analyzed by ANOVA).

It is well accepted that mitochondrion is a highly dynamic organelle and undergoes persistent mitochondria biogenesis, which refers to mitochondrion newborn, as well as mitophagy, which refers to mitochondrion clearance (Roger et al., 2017). We first tested the process of mitophagy both in cell model and animal model. We found that the expressions of PINK1 and Parkin, two central molecules initiating mitophagy (Bingol and Sheng, 2016), were both significantly elevated after HFD treatment in mice (Figure 3A) or PA treatment in LO2 cell (Figure 3B). This indicated that mitophagy was highly activated and may explain the decrease of mitochondrion quantity, which was further supported by the reduction of mitochondria DNA (mtDNA) (Figure 3C). PINK1 was only reversed by NAC in mice hepatic tissue but not in LO2 cells, indicating that different mechanisms may be involved. However, despite slight differences in possible mechanisms, NAC still maintained mtDNA level in HFD-treated mice (Figure 3C). Collectively, these data suggested that NAC can ameliorate excessive mitophagy induced by high fat both *in vivo* and *in vitro*, which may help maintain mitochondrion quantity.

Apart from mitophagy that represents the death of mitochondrion, mitochondria biogenesis that regulates the biogenesis of mitochondrion represents the supplement of the intracellular mitochondrion and is of importance to mitochondrial homeostasis. PGC1α is the key transcriptional factor coactivator of mitochondria biogenesis that regulates and stimulates the process. We then measured the expression of PGC1α and found that it was significantly downregulated by HFD (Figure 3A) or PA (Figure 3B) treatment, and NAC restored its expression. It was interesting to find that PA treatment not only decreased intrinsic PGC1α expression level but also interfered with PGC1α subcellular location as shown by immunofluorescence (Figure 3D) and Western blot of nuclear-cytoplasm separation (Figure 3F). In contrast, NAC reversed the abnormal subcellular location of PGC1α by significantly promoting its nuclear localization (Figure 3E). As the key transcriptional cofactor of mitochondria biogenesis, it is important for PGC1α to translocate into the nucleus and modulate subsequent transcriptional regulation of mitochondria biogenesis. Hence, NAC can promote mitochondria biogenesis and maintain mitochondrion quantity *via* promoting PGC1α nuclear localization.

Taken together, these data suggested that NAC on one hand prevented excessive mitophagy and on the other hand promoted mitochondria biogenesis to maintain mitochondrion quantity and help balance mitochondrion homeostasis.

### PGC1a Was the Key for NAC to Reduce Intracellular Lipid Accumulation

**FIGURE 4:**
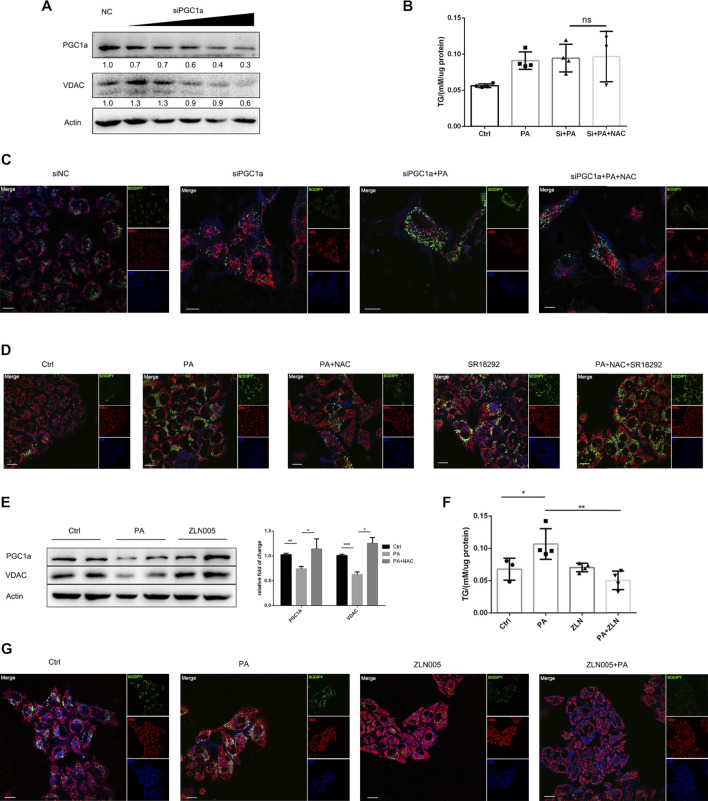
NAC reduced intracellular lipid accumulation *via* maintaining PGC1a level. **(A)** Representative Western blot image of PGC1a protein level after transfection with siRNA targeting at PGC1a; **(B)** intracellular TG concentration of cell in different groups; the concentration was adjusted to protein level; **(C,D)** BODIPY staining showed a different amount of lipid droplet in cells with PGC1a silenced **(C)** or pharmacologically inhibited **(D)**. The green channel was BODIPY, the red channel was mitochondria, and the blue channel was the endoplasmic reticulum (ER). Scale bar was 10 μm; **(E)** representative Western blot image of PGC1a and VDAC protein level after ZLN005 stimulation, a PGC1a specific agonist. The experiment was conducted triple; **(F)** intracellular TG concentration of cell in different groups; the concentration was adjusted to protein level; **(G)** BODIPY staining showed a different amount of lipid droplet in cells with ZLN005 treatment. The green channel was BODIPY, the red channel was mitochondria, and the blue channel was the ER. Scale bar was 10 μm (^*^ *p* < 0.05, ^***^ *p* < 0.001, and ^****^ *p* < 0.0001; all the data significance was analyzed by ANOVA).

Next, we were interested in whether the lipid-lowering effect of NAC was dependent on PGC1a. We firstly applied siRNA to silence the expression of PGC1a and found that the expression level of VDAC also decreased (Figure 4A). Interestingly, NAC no more exhibited lipid-lowering effect after PGC1a knockdown (Figures 4B,C). We also applied an antagonist targeting at PGC1a, SR18292 (Sharabi et al., 2017), and found that, after SR18292 treatment, NAC could not decrease intracellular lipid droplet as well. On the other hand, ZLN005, an agonist targeting at PGC1a (Zhang et al., 2013), could indeed upregulate the expression of PGC1a as well as VDAC (Figure 4E). ZLN005 treatment could alleviate PA-induced intracellular lipid accumulation (Figures 4F,G), indicating that activating PGC1a could reducing lipid amount, mimicking the effect of NAC**.**

### NAC Activated Sirt1 Deacetylase Activity to Promote PGC1a Nuclear Localization

**FIGURE 5:**
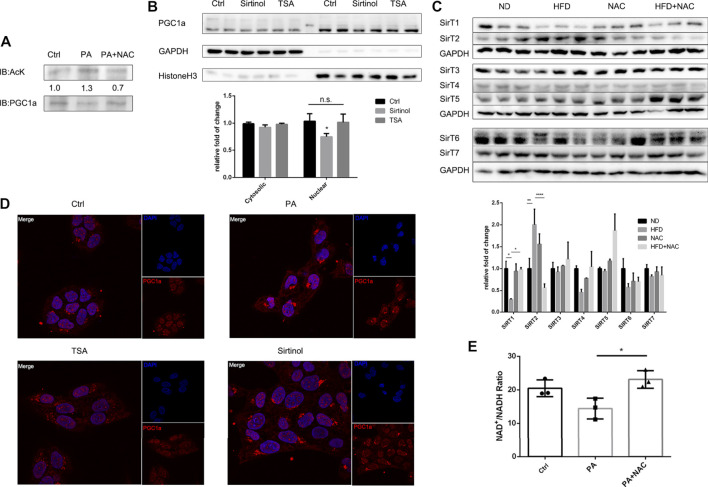
NAC activated Sirt1 to deacetylate PGC1a and maintained its mitochondria biogenesis activity. **(A)** Immunoprecipitation of PGC1a in LO2 cells underwent different treatment and Western blot analysis of acetylated lysine residue in immunoprecipitated PGC1a; **(B)** separation of nuclear and cytosolic protein under Sirtinol or TSA treatment and protein level of PGC1a measured by Western blot; **(C)** Western blot analysis of protein level of Sirtuin family in mice hepatic tissue; **(D)** immunofluorescence analysis of subcellular localization of PGC1a after TSA or Sirtinol treatment; scale bar was 10 μm; **(E)** NAD^+^/NADH ratio of LO2 cell after PA stimulation or NAC treatment. The experiment was conducted triple (^*^ *p* < 0.05; all the data significance was analyzed by ANOVA).

After confirming the important role of PGC1a in reducing intracellular triglyceride accumulation, we were interested in the mechanism behind it. It was reported that PGC1a underwent abundant posttranslation modification, including acetylation (Rodgers et al., 2005). We firstly accumulated PGC1a by immunoprecipitation and found that PA treatment significantly increased the acetylation level of PGC1a, and NAC treatment reversed hyperacetylation of PGC1a (Figure 5A). Next, we wondered which kind of deacetylase was responsible for PGC1a deacetylation. There are two main kinds of deacetylase in eukaryotic cells, Sirtuins and HDACs (Li et al., 2018). Hence, we used two antagonists, Sirtinol targeting at Sirtuin (Tsai et al., 2015) and TSA targeting at HDAC (Hu et al., 2013), and separated nuclear protein from cytoplasmic protein. It was found that only Sirtinol treatment could reduce nuclear PGC1a amount (Figure 5B). Immunofluorescence also revealed a similar result (Figure 5D). We also examined the protein expression of the Sirtuin family, Sirt1-7, in HFD-induced NAFLD mice hepatic tissues. We found that, in HFD treatment mice, Sirt1 was significantly downregulated, which could be reversed by NAC treatment (Figure 5C). Interestingly, Sirt2 was upregulated in HFD treatment mice, which NAC downregulated its expression, indicating a possible opposite role of Sirt2 against Sirt1 in regulating lipid metabolism. NAC could elevate the intracellular NAD+/NADH ratio (Figure 5E), which was thought to be a direct activator of Sirt1, and could partially explain the mechanism of the NAC lipid-lowering effect.

### NAC Could Treat HFD-Induced Hepatic Steatosis

In the previous animal research, NAC was gavaged prior to the beginning of HFD. However, it is of more translational value if NAC could treat hepatic steatosis rather than prevent it. We then fed the mice with HFD for 8 weeks and gavage the mice with NAC for the following 4 weeks. NAC could also reverse HFD-induced hepatic steatosis (Supplementary Figure S2A) and lower serum TG level (Supplementary Figure S2B). However, just consistent with the “prevention” research, NAC could not lower serum TC nor serum glucose level (Supplementary Figure S2B). NAC could also preserve the expression level of PGC1a and SirT1 (Supplementary Figure S2C), which further supports the role of SirT1/PGC1a in the treating effect of NAC in dealing with hepatic steatosis.

## Discussion

NAC is nowadays widely used in clinical work as a mucolytic agent to treat patients with too much sputum (Li et al., 2018a). However, it was reported that NAC can promote liver functions in NAFLD patients, and there are a number of clinical trials investigating the effect of NAC in treating metabolic syndromes (Oliveira et al., 2019). In this study, we demonstrated that NAC was effective in lowering triglyceride accumulation both *in vivo* and *in vitro*, and this effect is related to its ability to preserve mitochondrial function. NAC can be a promising drug in treating NAFLD clinically considering that several clinical trials supporting its effectiveness in dealing with NAFLD (Khoshbaten et al., 2010). And here we also provide evidences that NAC could treat HFD-induced NAFLD in mouse model. Mechanistically, we gave evidence that NAC could both inhibit excessive mitophagy and promote mitochondrial biogenesis, hence balancing mitochondrial function and preventing excessive ROS production. This may help promote clinical usage of NAC. Apart from NAFLD, NAC was also reported to be effective in treating obesity (Shen et al., 2018), ischemia-reperfusion injury of cardiomyocyte (Sivasinprasasn et al., 2019), and muscle atrophy (Wang et al., 2014b). Taken together, these reports, along with our data, indicate that NAC can be a useful agent to treat mitochondria dysfunction-related diseases.

Excessive fat intake is thought to be responsible for metabolic disorders nowadays. Liver is the major metabolic organ of the body, and numerous researches demonstrated that the Western diet or HFD could induce hepatic steatosis (Boden et al., 2015). Physiologically, diet lipid is oxidized by mitochondria through beta-oxidation. Excessive lipid accumulation, especially saturated fatty acid such as PA used in our study, was also reported to induce mitochondrial dysfunction, which further hinders lipid oxidation and intracellular accumulation (Alnahdi et al., 2019). Therefore, it is a reasonable strategy to preserve mitochondrial function to treat several metabolic diseases. Mitochondrion is an active cellular organelle and central power factory for cells. Once deteriorated, mitochondrion turns to be fragmental and initiates mitophagy to degrade damaged mitochondria, as well as stimulating mitochondrial biogenesis to maintain a healthy mitochondria pool. Although PINK1/Parkin upregulation is a classical mitophagy initiation signal (Wang et al., 2019), recent studies also indicate PINK1/Parkin-independent mitophagy initiation mechanisms such as AMPK-dependent TBK1 activation (Villa et al., 2018). These reports indicate that the intracellular energy state is tightly related to the mitophagy process. In our study, we found that the mitophagy was actively initiated both *in vivo* and *in vitro*, and NAC could reverse this process. In further studies, it is interesting to test whether NAC could ameliorate mitophagy in PINK1/Parkin-independent pathway.

As a central organelle of cell catabolism and metabolism, maintaining enough mitochondria quantality is vital to the cell. Mitochondrial biogenesis is under rigorous regulation of PGC1a, and in our work, we gave evidence that PGC1a was the key for NAC to exhibit lipid-lowering effect, and this was related to Sirt1 deacetylase. Deacetylation of PGC1a was shown to be dependent on Sirt1 activity, which was provoked by the intracellular NAD^+^/NADH ratio. Sirt1 was reported to be involved in calorific restriction and energy metabolism regulation, and an underlying connection between Sirt1 and AMPK was also reported (Tian et al., 2019). As important energy state sensors, Sirt1 and AMPK showed close interplay in regulating mitochondria function. However, while some reports indicate that AMPK acts as an upstream regulator of Sirt1 and PGC1a, there were also contradictory results (Park et al., 2012). It is possible that different cell/tissue types and different nutrient conditions may account for this divergency.

It is interesting to find that posttranslation modification of certain proteins has a tremendous effect on nutrition metabolisms. Here, we have shown that posttranslation acetylation of PGC1a also has an effect on lipid metabolism by affecting mitobiogenesis. Rodgers et al. reported that PGC1a was abundant of lysine-acetylated by Sirt1 and modulating mitochondrial biogenesis (Rodgers et al., 2005). Here, we also found that PGC1a was hyperacetylated after PA treatment and is consistent with previous researches. However, we did not identify the exact acetylated amino acid residue and this needs further investigation. It was reported that PGC1a can act as a coactivator of vast transcription factors and have an RNA recognition motif (RRM) at its C terminal (Sano et al., 2007), covering one acetylation site Lys^758^. Hence, it is possible that hyperacetylation of Lys^758^ may alter RRM conformation and affect the PGC1a function.

Sirt1 is an important deacetylase that regulates lipogenesis and fatty acid oxidation by deacetylating numerous metabolic-related transcription factors including PPARa (Kalliora et al., 2019), Lipin1 (Li et al., 2018b), and PGC1a (Tsai et al., 2018). Sirt1 is a nuclear-localized enzyme and can sense intracellular NAD^+^/NADH ratio, which is a direct activator of Sirt1 (Jang et al., 2012). It was reported that the NAD^+^/NADH ratio was downregulated in the process of NAFLD (Park et al., 2016), and this is consistent with our observation. In this study, we have shown that NAC can not only restore Sirt1 protein level but also maintain NAD^+^/NADH ratio and hence restore Sirt1 activity. It makes sense that PGC1a can be deacetylated by Sirt1 and promoting mitobiogenesis by NAC.

Mitochondrion is the major source of intracellular ROS production. It is well-known that excessive ROS production can oxidase lipid and attack DNA, further causing cell death in many metabolic diseases (Włodarczyk and Nowicka, 2019). Controlling ROS level is an important strategy to treat metabolic diseases (Włodarczyk and Nowicka, 2019). Due to the pharmacological character of NAC, it can antagonize ROS free radical electron by its sulfhydryl and acting as an anti-ROS agent. In this study, we confirmed the ROS-scavenging role of NAC, as it could decrease ROS and MDA level *in vivo* and ROS level *in vitro*. We also measured intrinsic antioxidant enzymes such as SOD1 and SOD2 and found that although HFD did not significantly reduce enzyme expressions, NAC treatment could significantly augment the SOD1 level. This indicating that NAC not only antagonizes ROS by its pharmacological feature but also increases antioxidant enzymes to exhibit ROS scavenge effect. On the other hand, the exact resource of ROS is still unrevealed in our work, since no significant changes were found among NOX1, NOX2, and NOX4 (Long et al., 2017). However, considering the importance of mitochondria in producing ROS, it is still possible that other oxidases locating at mitochondria are responsible for the excessive production of ROS in the development of NAFLD.

However, there are several limitations of our study. First, we did not test the optimal dose to treat NAFLD in the murine model. It is important to titer the optimal dose in future studies, while in our study, we proved that 150 mg/kg in male C57Bl/6 mice may be effective. Another major limitation of our study is that we only conducted an animal experiment in male mice. Recently, different responses between males and females to energy disorder and NAFLD are reported (Yoshioka et al., 2020), and this is considered to be related to sex hormones, age, and other reproductive elements (Lonardo et al., 2019). In future researches, more attention should be paid to female animal models, as well as in clinical trials. While we have suggested that preserving mitochondrial function *via* SirT1/PGC1a signaling pathway is vital to the lipid-lowering effect of NAC, we still cannot exclude other possible involved mechanisms such as interfering entry of fatty acids or *de novo* lipogenesis in liver, and excessive fatty acid could trigger endoplasmic reticulum (ER) stress, which is a typical pathological process participating in the development of NAFLD. Future investigation can further explore the possible mechanisms regarding the lipid-lowering effect of NAC.

In conclusion, NAC can prevent and treat high-fat-induced NAFLD in a murine model by preserving mitochondria function, hence improving mitobiogenesis and preventing excessive mitophagy. NAC can restore the ratio of NAD^+^/NADH to maintain Sirt1 activity, which is the key deacetylase to keep PGC1a from high-fat-induced hyperacetylation. Activation of the Sirt1-PGC1a axis promotes mitochondria function and inhibits ROS production to protect the liver from lipid toxicity. Carrying out clinical trials may be considered to test the ability NAC of to treat NAFLD.

## Data Availability

The raw data supporting the conclusion of this article will be made available by the authors, without undue reservation.

## Supplementary Material

Figure Supplementary S1 NAC treatment upregulated the expression level of antioxidant enzyme and triglyceride metabolism enzymes but showed little effect on NOXs. **(A)** Immunoblot of SOD1 and SOD2 in hepatic tissue of model mice and quantification of Western blot. **(B)** Immunoblot of SREBP1c, SREBP2, and LPL in hepatic tissue of model mice and quantification of Western blot. **(C)** Immunoblot of NOX1, NOX2, and NOX4 in hepatic tissue of model mice and quantification of Western blot. **(D)** Immunohistochemistry of NOX4 in the liver section of mice; the scale bar is 50 μm (^*^ *p* < 0.05; ***p* < 0.01; all the data significance was analyzed by ANOVA).

Figure Supplementary S2 NAC could treat HFD-induced hepatic steatosis. **(A)** HE staining of mice liver section in different groups. Mice were firstly fed with 8 weeks of HFD and then receive 4 weeks of NAC gavage; the scale bar is 50 μm. **(B)** Serum TG, TC, and glucose level of different mice. **(C)** Immunoblot of PGC1a and SirT1 in hepatic tissue of model mice and quantification of Western blot (^*^ *p* < 0.05; ***p* < 0.01; all the data significance was analyzed by ANOVA).
