# Supplementary figures and images for: N-Acetyl Cysteine Ameliorates High-Fat Diet-Induced Nonalcoholic Fatty Liver Disease and Intracellular Triglyceride Accumulation by Preserving Mitochondrial Function
Source: Front Pharmacol. 2021 Sep 13;12:636204. doi: 10.3389/fphar.2021.636204 (PMC8473737; doi:10.3389/fphar.2021.636204)

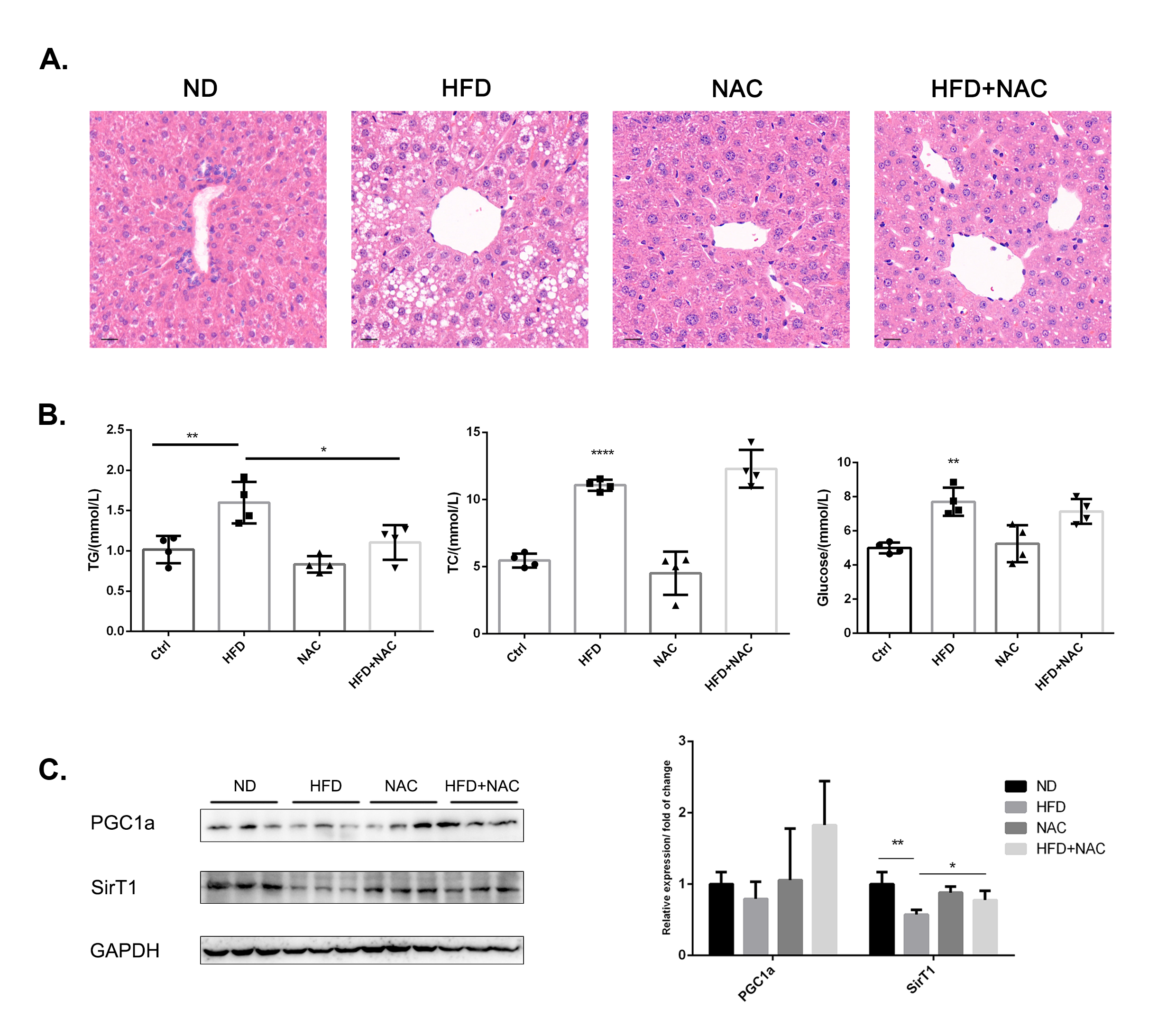

Supplement: Supplementary file 1 [file Image2.TIF]

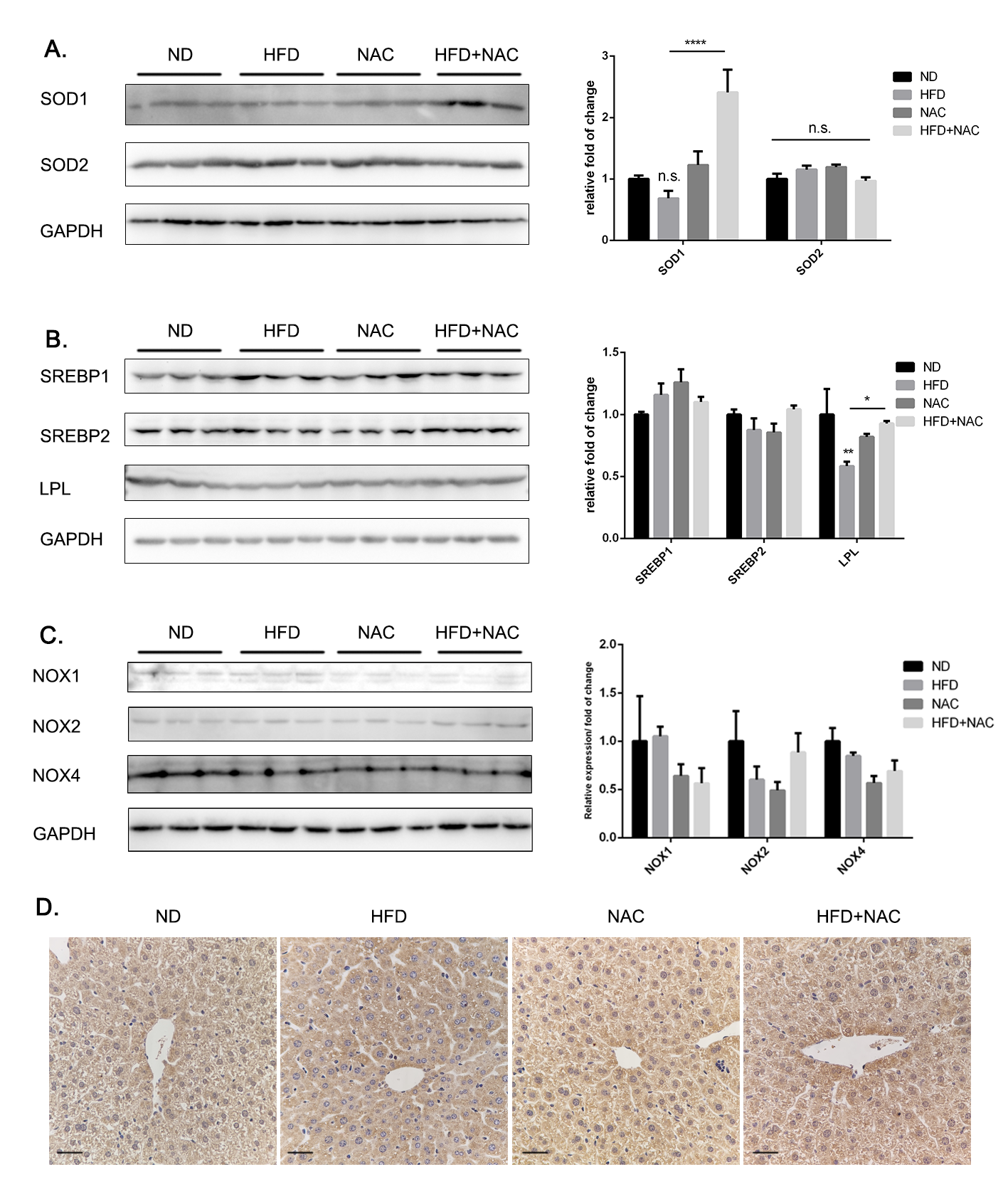

Supplement: Supplementary file 2 [file Image1.TIF]
